# Mapping allosteric pathway in NIa‐Pro using computational approach

**DOI:** 10.15302/J-QB-022-0296

**Published:** 2023-03-01

**Authors:** Rashmi Panigrahi, Senthilkumar Kailasam

**Affiliations:** ^1^ Department of Biochemistry University of Alberta Edmonton T6G 2H7 Canada; ^2^ Canadian Centre for Computational Genomics McGill University Montréal H3A 0G1 Canada

**Keywords:** NIa‐Pro, VPg, simulation, residue interaction network, allostery

## Abstract

**Background:**

Computer simulation studies complement *in vitro* experiments and provide avenue to understand allosteric regulation in the absence of other molecular viewing techniques. Molecular dynamics captures internal motion within the protein and enables tracing the communication path between a catalytic site and a distal allosteric site. In this article, we have identified the communication pathway between the viral protein genome linked (VPg) binding region and catalytic active site in nuclear inclusion protein‐a protease (NIa‐Pro).

**Methods:**

Molecular dynamics followed by *in silico* analyses have been used to map the allosteric pathway.

**Results:**

This study delineates the residue interaction network involved in allosteric regulation of NIa‐Pro activity by VPg. Simulation studies indicate that point mutations in the VPg interaction interface of NIa‐Pro lead to disruption in these networks and change the orientation of catalytic residues. His142Ala and His167Ala mutations do not show a substantial change in the overall protease structure, but rather in the residue interaction network and catalytic site geometry.

**Conclusion:**

Our mutagenic study delineates the allosteric pathway and facilitates the understanding of the modulation of NIa‐Pro activity on a molecular level in the absence of the structure of its complex with the known regulator VPg. Additionally, our *in silico* analysis explains the molecular concepts and highlights the dynamics behind the previously reported wet lab study findings.

## Abstract 2

**Author summary:** Allosteric control of enzymes is a key regulatory process, ubiquitous in all kingdoms of life. It involves the binding of the specific regulatory molecule (either an activator or an inhibitor) to a location distal from the enzyme’s active site. Our research provides a simple *in silico* methodology for gaining insight into the allosteric pathways that regulate specific enzyme activity. In the presence of a 3D structure of the enzyme and the knowledge of specific interaction sites with its regulator, this methodology can be applied to understand enzyme regulation, even in the absence of the structure of the enzyme‐regulator complex.

## INTRODUCTION

Potyviruses are the largest genus of RNA viruses that infect crop plants, making them concerns to farmers worldwide [1]. The pepper vein banding virus (PVBV) belongs to the potyvirus group and infects Solanaceae plants, such as chilli and capsicum. It also encodes for a large 340‒370 kDa polyprotein [2]. The 5′ terminus of the genomic RNA is covalently associated via a phosphodiester bond to the viral protein genome linked (VPg) [3]. VPg acts as a primer for the initiation of positive and negative strand RNA synthesis during viral replication [4]. The polyprotein is subsequently cleaved by three proteinases, namely P1‐protease (P1‐Pro), Helper component‐protease (HC‐Pro), and nuclear inclusion protein‐a protease (NIa‐Pro), all of which are encoded in the virus. This proteolytic cleavage, or polyprotein processing, occurs after translation at different stages of the viral life cycle [5]. NIa‐Pro is a serine‐like cysteine proteinase with an active site made up of a catalytic triad consisting of His46, Asp81, and Cys151 [3,6,7]. This proteinase recognizes heptapeptide sequences and cleaves at Gln/Ala, Gln/Ser, or Gln/Thr with amino acids flanking at either side of the cleavage site. Contrary to P1‐Pro and HC‐Pro that demonstrate cis activity only, NIa‐Pro has both *cis* and *trans* cleavage activity [8,9]. P1‐Pro and HC‐Pro cleave at their own C‐termini. However, NIa‐Pro can cleave at seven different sites along the translated polyprotein [10]. One such *cis* cleavage site exists between VPg and NIa‐Pro, which is known to be suboptimal [11] as a glutamate is found in the P1 position of the recognition sequence instead of a glutamine. Any mutation at the cleavage site leads to defective viral replication [11,12]. Therefore NIa‐Pro exists as VPg‐Pro, with an N‐terminal VPg and C‐terminal NIa‐Pro. VPg is an intrinsically disordered protein and acts as a protein interaction hub [13,14]. It harbors the walker motifs A and B, which is functional only upon interaction with NIa‐Pro [14]. Furthermore, the deletion of the N terminal residues of VPg led to a decrease in protease activity as the interaction of VPg with NIa‐Pro is impaired [13]. Surface plasmon resonance studies have shown that the affinity of VPg for NIa‐Pro is drastically decreased upon deletion of 113 residues from the N‐terminus of VPg. Additionally, enzyme kinetic analyses have shown that VPg increases enzymatic turnover when associated with NIa‐Pro [15]. The increase in protease activity of NIa during interaction with VPg can be described as an allosteric event.

Allosteric regulation, in which the perturbation at one site of the protein affects the distal catalytic site, is a widely observed phenomenon [16]. This mechanism helps to regulate the activity and dynamic adaptability of proteins while they perform their functions [17]. Protein structures have evolved in the constraint of their designed functions and need to be robust against mutations [18,19]. Hence, establishing good communication paths between distal sites and the catalytic site of the protein bring about the precision in function. Some of these perturbations may not induce visible conformational changes; however, they do affect the catalytic site [20,21].

Allosteric communications are best described by residue‐residue interaction networks. Network models that give rise to a high degree of interaction cooperativity based on three‐dimensional structure have been successfully used to delineate mode of allosteric communication on a molecular level [22, 23, 24]. Elucidating these communication networks are challenging as they involve multiple modal communication pathways between the binding site and distal active site. These trajectories are not ascertainable by a single crystal structure, which provides a snapshot of the interactions [23]. Hence, theoretical approaches, such as molecular dynamics (MD) simulation studies are precious tools used in obtaining an atomistic insight of the above phenomenon over a time period [25]. The comparative analysis of specific patterns of inter‐residue fluctuations can aid in identifying plausible mechanisms of coordinated conformational changes in the protein due to specific mutations or binding of the allosteric partner. The shortest path is identified as the path in which two distal site residues are connected by the minimal number of intermediate residues. These intermediate residues are centrally conserved and are known as conserved interconnectivity determinants (CICD). Mutations in these residues can cause changes in the optimal communication pathway [18], leading to a disruption of protein function. This explains why experimental evidences suggest that a number of single site mutants have little or no impact on the protein function, whereas mutations in key residues perturb the function to a larger extent [18,26].

The current study was carried out with an aim to delineate the communication pathway in NIa protease between its VPg binding site and the catalytic site. Enzyme kinetics have shown that Ser129, a surface exposed residue on NIa‐Pro, is essential for enzyme activity, despite it being located 16.9 Å from the Cys151 Cα (catalytic cysteine) [15]. This serine is known to be phosphorylated by a host cell kinase. NIa‐Pro Ser129Asp (phospho‐mimetic mutant) demonstrate a drastic loss in activity compared to the wild type as observed during enzymatic analysis, suggesting that this phosphorylation could be regulating the protease activity of NIa‐Pro [15]. This suggests that changes in the distal Ser129 is being relayed to the active site through a residue‐to‐residue interaction pathway.

In addition, Ser129 lies in close proximity to Trp143, which lies distal to the Cys151 (14.47 Å apart). It had been shown using fluorescence studies that Trp143 is present on the VPg binding interface of NIa‐Pro [15]. Furthermore, the mutation of this tryptophan to alanine did not affect the binding of the substrate, but rather abrogated the cleavage reaction and the subsequent product release. Simulation studies have shown that the Trp143Ala mutation altered the orientation of Cys151 (causing it to move away from the catalytic residues His46 and Asp81) [15]. The WC loop that connects Trp143 with Cys151 was postulated to be crucial for the substrate‐product interaction [15,27,28]. It has also been speculated that the binding of VPg on the face of NIa‐Pro containing Trp143 might influence the structural integrity of the WC loop, thus aid in the regulation of catalytic activity. Trp143 is connected to Cys151 via noncovalent interactions mediated by His142 and His167. These residues are conserved across all members of the *Potyvirus* genus [15].

In order to investigate allostery on a molecular level in NIa protease and its mutants, MD simulation studies followed by analysis of residue interaction networks (RIN) have been undertaken. The comparison of allosteric communication pathways between different mutants elucidated in this study highlights the diverse means adopted by the protease mutants to perform their enzymatic function. In the absence of a three‐dimensional model of VPg and/or NIa‐VPg complex, our findings elucidate the communication pathway and key hub residues between the VPg binding site, as well as the catalytic site and key hub residues of NIa protease, thus mapping the residue‐residue interaction network that might be involved in the allosteric regulation of NIa‐Pro.

## RESULTS

### Analysis of trajectory

**Figure 1:**
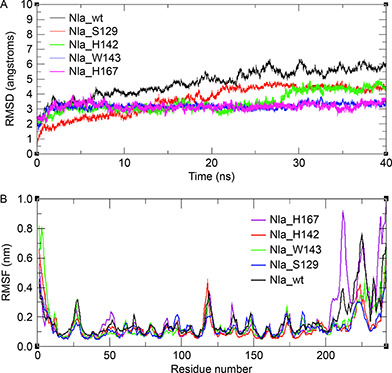
**Analysis of MD simulation trajectories for NIa‐Pro and the mutants.** (A) Root mean square deviation (RMSD) plots for all the systems. (B) The atomic positional fluctuation (RMSF) plots obtained for each of the above system.

In order to gain molecular level insight into the residue‐residue interaction network of NIa‐Pro and its mutants, MD simulation studies have been undertaken *.* The results provide an in‐depth understanding of the structure and dynamics of these proteins. I‐TASSER was used to generate the best model of NIa protease for this study. NIa protease from PVBV shares a sequence identity of 47% and 45% with respect to the TEV protease and TVMV protease, respectively. The RMSD between the model generated using ITASSER and the crystal structure of TEV protease (PDB: 1LVM) was 0.90 Å, while that with the crystal structure of TVMV protease was 1.60 Å. Furthermore, the Ramachandran plot analyses showed that 90% residues were present in the favored regions (Supplementary Fig. S1). The RMSD of the Cα atoms for each simulation was calculated using the starting structure as the reference structure to assess the overall stability ( Fig.1). The RMSD values were found to be 4.76 Å, 3.60 Å, 3.42 Å, 3.18 Å, and 3.15 Å for NIa_wt, NIa_S129, NIa_H142, NIa_W143, and NIa_H167 systems, respectively. The RMSD values of the mutants were lower compared to the wild type, which correlates with the previously reported findings [15]. An analysis of Cα atomic fluctuations for each of the systems showed that the N and C termini exhibited higher RMSF compared to other regions. The C‐terminal that comprises of 200−242 residues demonstrated extensive fluctuations in NIa_wt and NIa_H167 systems ( Fig.1). NIa‐Pro wild type and H167A mutant have 35 hydrogen bonds in each of their systems. The S129A, W143A, and H142A mutants have similar quantities of hydrogen bonds *i.e.*, 44, 43, and 41, respectively. The total number of salt bridges in NIa‐Pro and H142A mutant is 16 and 19, respectively. The S129A, W143A, and H167A mutants have similar quantities of salt bridges *i.e*., 23, 23, and 22, respectively.

### Essential dynamics

**Figure 2:**
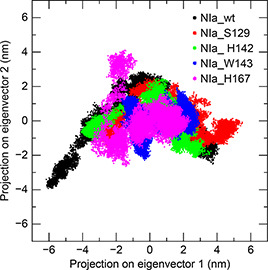
Essential dynamics—2D projection of individual trajectories with their first 2 eigenvectors: vector 1 and vector 2.

The harmonic and large‐scale motions in various systems occurring in the essential subspace were analyzed using principal component analysis. The overall motions observed in each system were decomposed into eigenvectors. The first vector accounted for the majority (>60%) of the total movement in all the systems. The conformation sampled in the subspace spanned by the first two eigenvectors was visualized using two‐dimensional projection plots designed for all trajectories ( Fig.2). The conformational space sampled was found to be diffused over a larger area in the wild type compared to the mutants. However, multiple local minima were observed in all systems. In the case of the NIa_W143 system, the conformational spread was comparatively restricted and the number of local minima was reduced.

**Figure 3:**
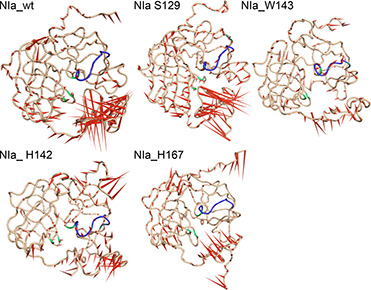
**Porcupine plots for the wild type and the mutant systems are presented in a cone model.** The length and direction of the cone (red) represents the magnitude and direction of motion.

The direction and magnitude of movement captured by the first eigenvector in all systems were displayed using porcupine plots ( Fig.3). The N and C terminal regions of the proteins in all the systems showed higher motion compared to other regions. In the case of the wild type, there was random movement observed in the region encompassing residues 206−242, as observed in the rms fluctuations. Similar movement was observed in the NIa_S129 system. In the NIa_H142 system, the motions were relatively reduced, while the C‐terminal seemed to move away from the protein core in the NIa_H167 system. Interestingly, the porcupine plot showed relatively restricted movement of the C‐terminal region in the NIa_W143 system.

### Residue interaction networks

Different interaction networks for communication between the VPg binding site and catalytic site were evaluated using the last 5 ns averaged structures of MD trajectories. A comparative analysis between the RINs from the mutant systems and that of the wild type showed interesting differences. In NIa_wt, the WC loop and several other noncovalent interaction networks mediated the communication from Trp143 to Cys151. The shortest noncovalent interaction network was Trp143‐His142‐His167‐Cys151 (Supplementary Fig. S2). This network was distorted and the position of the catalytic site residues and/or their side chains were affected and are discussed below.

#### NIa_S129 system

**Figure 4:**
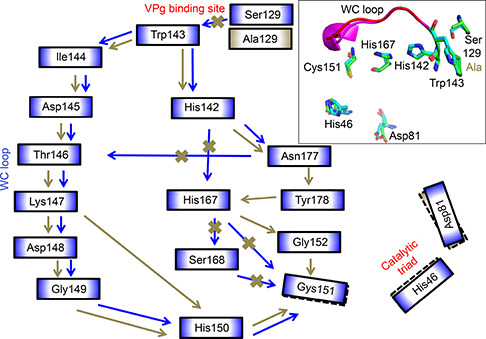
**Schematic diagram showing the residue interaction networks in the NIa‐Pro wild type and NIa_S129 system.** The inset shows the overlay of WC loop conformations (wild type: magenta, and mutant: red), orientations of the catalytic triad along with the two crucial residues, His142 and His167 (wild type: green sticks, and mutant: cyan). The residue communication pathway from the VPg interaction interface to the catalytic site for the wild type is shown using blue arrows. The modified pathway with the Ser129Ala mutation is shown using golden green arrows. The native communication pathway between the two residues that is no longer effective in the mutant is marked by golden green crosses. The communication pathway is based on hydrogen bonding, hydrophobic, and main‐chain interactions, which is also demonstrated in ligplot in the supplemental.

With the mutation of Ser129 to alanine, the side chain interaction with Trp143 is lost. A slight displacement of the side chains of the three active site residues was observed ( Fig.4 and Supplementary Fig. S3). The shortest communication pathway was Trp143‐His142‐Asn177‐Try178‐His167‐Gly152‐Cys151.

#### NIa_W143 system

**Figure 5:**
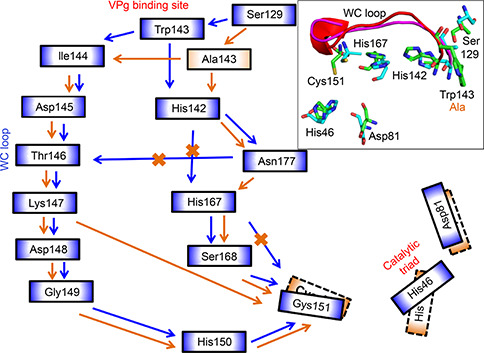
**Schematic diagram showing the residue interaction networks in the NIa_W143 system with reference to the wild type.** The inset shows the overlay of WC loop conformations (wild type: magenta, and mutant: red), orientations of the catalytic triad (wild type: green sticks, and mutant: cyan), and the two crucial residues, His142 and His167 (wild type: green sticks, and mutant: cyan). The residue communication pathway from the VPg interaction interface to the catalytic site for the wild type is shown using blue arrows. The modified pathway due to the Trp143Ala mutation is shown using orange arrows. The native communication pathway between the two residues that is no longer effective in the mutant is marked by orange crosses. The communication pathway is based on hydrogen bonding, hydrophobic, and main‐chain interactions, which is also demonstrated in ligplot in the supplemental.

When W143 was mutated to alanine, flipping of the side chain of Cys151 was observed. The imidazole ring of His46 was orientated into the active site pocket ( Fig.5 and Supplementary Fig. S4). Although the side chain of Trp143 was lost with the mutation to alanine, the interaction with Ser129 was maintained. However, a displacement of His142 and a subsequent rearrangement of the interaction with His167 through Asn177 were observed. Thus, the shortest pathway noted was Ala143‐His142‐Asn177‐His167‐Cys151. The side chain of Cys151 flipped away from the active site pocket and the imidazole ring of His46 faced into the pocket.

#### NIa_H142 system

**Figure 6:**
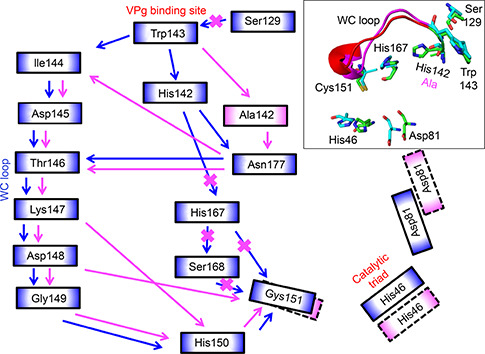
**Schematic diagram showing the residue interaction networks in the NIa_H142 system with reference to the wild type.** The inset shows the overlay of WC loop conformations (wild type: magenta, and mutant: red), orientations of the catalytic triad, and the two crucial residues, His142 and His167 (wild type: green sticks, and mutant: cyan). The residue communication pathway from the VPg interaction interface to the catalytic site for the wild type is shown using blue arrows. The modified pathway due to the His142Ala mutation is shown using pink arrows. The native communication pathway between the two residues that is no longer effective in the mutant is marked by orange crosses. The communication pathway is based on hydrogen bonding, hydrophobic, and main‐chain interactions, which is also demonstrated in ligplot in the supplemental

When His142 was mutated to alanine, a flip in the side chain of Cys151 was observed and the two catalytic residues His46 and Asp81 were displaced ( Fig.6 and Supplementary Fig. S5). Furthermore, the shortest interaction network observed in the wild type was disrupted and additional pathways were established with the involvement of Asn177 and the residues of the WC loop. Additionally, the noncovalent interaction between Trp143 and Ser129 was disrupted because of the change in orientation in the Ser129. Moreover, the new interaction networks in NIa‐Pro (H142A) and NIa‐Pro (H167A) are longer than the non‐covalent interaction pathway (Trp143‐His142‐His167‐Cys151) in wild type NIa‐Pro. The conformation of the WC loop is different in His142Ala NIa‐Pro compared to the wild type ( Fig.6).

#### NIa_H167 system

**Figure 7:**
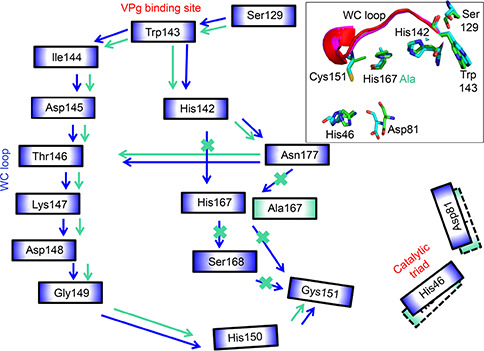
**Schematic diagram showing the residue interaction networks in the NIa_H167 system with reference to the wild type.** The inset shows the overlay of WC loop conformations (wild type: magenta, and mutant: red), orientations of the catalytic triad, and the two crucial residues, His142 and His167 (wild type: green sticks, and mutant: cyan). The residue communication pathway from the VPg interaction interface to the catalytic site for the wild type is shown using blue arrows. The modified pathway due to the His167Ala mutation is shown using green arrows. The native communication pathway between the two residues that is no longer effective in the mutant is marked by orange crosses. The communication pathway is based on hydrogen bonding, hydrophobic, and main chain interactions, which is also demonstrated in ligplot in the supplemental.

For the system where His167 was mutated to alanine, although the interaction between Ser129 and Trp143 was maintained, the interactions involved in the shortest pathway observed in the wild type were disrupted due to absence of side chain interactions of A167 ( Fig.7 and Supplementary Fig. S6). Furthermore, a slight displacement of Asp81 and orientation change of the imidazole ring of His46 was noted. Therefore, another pathway from Trp143 involving WC loop residues exists (Trp143‐His142‐Asn177‐Thr146‐Lys147‐Asp148‐Gly149‐His150‐Cys151).

## DISCUSSION

Understanding allosteric regulation in proteins have opened doors for the design of allosteric modulators of therapeutically important targets [29,30]. This is due to the improved selectivity offered by allosteric sites in contrast to active sites. Perturbation at these allosteric sites induced by mutation [31], post‐translational modifications [32], ligand binding, etc. can induce changes in catalytic activity [33], structure [34], or oligomeric state [35]. The traditional method of mapping allosteric sites involves the creation of mutants or deletion of specific amino acids stretches distal to the catalytic site, followed by subsequent analysis of protein activity. An upregulation or downregulation of activity indicates the involvement of mutated residue(s) in allosteric regulation [36,37]. Directed evolution and high throughput screening methods have also been used [38]. These methods are limited by enzyme signal detection. After the experimental validation of allostery, molecular dynamics (MD) simulations have been performed with either a docked or crystal structure of the complex. The simulation studies capture the binding induced conformational changes and the change in free energy is calculated using MMPB/GBSA algorithms. In the last decade, computational approaches have provided the inexpensive first step to predict such allosteric sites, trace crucial residues in the allosteric pathway, validate experimental findings, and design small molecules as drug candidates. For instance, tools like AlloPred [39] and PARS [40] use normal mode analysis to predict allosteric pockets on proteins. The dynamics upon the binding of modulators are modelled alongside support vector machine algorithms (in AlloPred) and structural conservation scores (in PARS). As the allosteric type and pathways are controlled by forces imposed upon ligand binding to the target protein, a prediction method named AlloType [41] has been recently developed. In this method, coarse grained anisotropic network model is used to define the response of the protein structures. Structural changes due to ligand binding are described using linear response theory. The free energy changes along with conformational changes determine the allosteric coupling strength. Several comprehensive allosteric data collections have been curated, including Allosteric Databases (containing protein structures and modulators) and AlloMAPS (containing allosteric mutants) [42]. These databases have aided the development of allosteric drug design platforms, including AlloSite [43,44], CavityPlus [45], PARS, AlloSigMA for allosteric residue analysis [46,47], and AlloFinder for identification of modulators [48]. Allosteric coupling networks can be deduced from pairwise interactions at residue level, revealing densely connected elements. Molecular dynamics has been commonly used to trace such allosteric networks [49,50]. Simulation studies of large proteins can be computationally expensive. In such cases, fluctuation dynamics study using elastic network model (ENM) with normal mode analysis using single parameter harmonic potential has provided success [51, 52, 53]. The residues were considered as the nodes and linkers of the pathway and are the inter‐residue potentials stabilizing the folded state. A key limitation in ENM is the presence of cutoff distance. Ohm is a platform for mapping allosteric communication and is based on the perturbation propagation algorithm. It predicts allosteric sites, inter‐residue correlations, and communication pathways [54]. A combination of graph theory‐based methods like bond‐bond propensity analysis and Markov transient analysis have been successfully used to map the allosteric communication pathway in proteins, including the main protease of severe respiratory syndrome coronavirus 2 [55,56]. Recently, Duan *et al*. wrote a comprehensive review, which is an excellent read on the advancement of computational methods applied to understand allostery [42]. Thus, various computational strategies have been promising in our journey to understanding allostery in various proteins, including apoptosis related protein, Caspase1 [54], the regulator protein CheY [54], ClpY ATPase [57], Hsp90 [58], monoacylglycerol lipase [19], G protein coupled receptors [59], etc.

In PVBV, the binding of VPg regulates the catalytic function of NIa‐Pro as evidenced by enzyme kinetics and binding studies [13]. The molecular details underlying this communication pathway was poorly understood. VPg is unstructured and its residues involved in its binding to NIa‐Pro, along with the conformational changes that might occur due to this interaction, are yet to be studied. In the absence of the complex structure, we use traditional mutation studies to shed light on the communication pathway. In this study, we provide molecular insights into the allosteric regulation of NIa‐Pro and the residue interaction network between the VPg interacting interface and catalytic site of NIa‐Pro. Our analysis from MD studies shows significant correlation with previously documented biochemical studies [13]. The biochemical experiments suggest that the protease activity of the NIa‐Pro mutants, Ser129Ala, Trp143Ala, His142Ala, and His167Ala are impaired compared to the wild type [60]. Our simulation studies indicate that the decrease in catalytic activity of the mutants could possibly be associated with a conformational change in the catalytic triad. The point mutations Ser129Ala and Trp143Ala are located distal from the catalytic triad. However, these mutations affect the enzymatic activity as the optimal residue interaction pathway is disrupted. In the case of the NIa_S129 system, the short interaction network involves His142 and His167, along with other residues. This finding is similar to that of the NIa_W143 system; however, distortion in the side chains of Cys151 and His46 was observed. Interestingly, the residue interaction networks of the NIa_H167 and NIa_H142 systems are longer and involve the residues of the WC loop. Furthermore, the orientations of catalytic site residues are different from that in the wild type. The changes in the orientation of the residues of the catalytic triad are associated with the activity of NIa‐Pro. Our findings delineate the residue level communication pathway between Trp143 present on the VPg interaction interface and the catalytic site. The residues operate in a concerted manner and aid the allosteric regulation the NIa‐Pro. In case a VPg‐NIa‐Pro complex can be obtained in the future using any experimental technique, simulation studies on the complex would reveal more details and add to the current study.

## MATERIALS AND METHODS

### Starting structures

The sequences of NIa‐Pro (accession number NP_188424, At3g17970) was used for the study. In order to obtain a reliable model for *in silico* studies, the protein sequence was submitted for automated protein structure modelling using the I‐TASSER pipeline. This server builds 3D models based on multiple threading alignments and iterative structure assembly simulations [61, 62, 63, 64]. Since the above method used multiple threading alignments, there is minimal bias towards a particular structural model as in the case of homology modelling [65,66]. The model systems for the mutants were built using the coordinates of the wild type obtained from the I‐TASSER server as templates. The wild type and mutant starting structures were further validated using Ramachandran plots [67].

Six systems were prepared: NIa wild type (NIa_wt), NIa‐Pro Ser129Ala mutant (NIa_S129), NIa‐Pro His142Ala mutant (NIa_H142), NIa‐Pro Trp143Ala mutant (NIa_W143), and NIa‐Pro His167Ala mutant (NIa_H167).

### Preparation

The simulation studies were performed using assisted model building with energy refinement (AMBER) suite version 12 [68] associated with the all‐atom ff12SB force field [69,70]. The starting structures for the simulations were neutralized using Na^+^ and Cl^−^ ions. The hydrogen atoms were positioned using the *tleap* module from AMBERTOOLS12. The protein was centered in a solvent truncated octahedron box, which was comprised of TIP3P (3‐point charged) triangulated water molecules with a 12 Å cut off in all directions [71]. The total number of atoms including water molecules was approximately 20,000 across each of the various systems. The systems were minimized using a two‐phase energy minimization procedure, which included 2,500 cycles of steepest descent and 2,500 cycles of conjugate gradient with solute atoms restrained by a harmonic potential with a constant force of 50 kcal mol^−1^ Å^2^. This was followed by 5,000 steps of unrestrained whole system minimization. 50 ps of density equilibration with weak harmonic restraints (2 kcal mol^−1^ Å^2^) on the solute molecule was performed followed by unrestrained equilibration for 500 ps using under constant pressure and temperature conditions. All simulations ran with constraints using the SHAKE algorithm [72] on hydrogen‐linked bonds with a tolerance of 0.0001. To evaluate long‐range electrostatic interactions, the Particle Mesh Ewald (PME) method [73] was used with a 9 Å cut‐off. A 2 fs integration time step was used to numerically solve Newton’s equations of motion. Langevin dynamics was used to maintain a constant temperature of 300 K throughout the simulations. All the simulations were carried out using the PMEMD module in AMBER12. Production runs were performed for 50 ns in an explicit solvent environment and isothermal‐isobaric (NPT) ensemble.

### Analysis

After 40 ns of production run, each of the trajectories was analyzed based on the variation in kinetic and potential energies under the NPT ensemble using the *ptraj* program. The root‐mean‐square deviation (RMSD) and atomic positional fluctuation per residue (RMSF) were analyzed to understand the overall conformational change throughout the trajectory. Hydrogen bonding, hydrophobic interactions, and their occupancies were calculated using HBPLUS using the criteria described by McDonald and Thornton for the definition of a hydrogen bond [74]. Only the hydrogen bonds with occupancies above 50% were considered for analysis. LIGPLOT was used to map the hydrogen and hydrophobic bonding patterns between the WC loop residues, mutated residues, and other residues of the NIa protease in various systems [75]. The last 5 ns averaged structures from each simulation were used for further analysis. Visual molecular dynamics (VMD) was used for visualizing the trajectories of the simulations of the six systems [76]. Furthermore, performing principal component analysis (PCA) substantiated the above results [77]. Collective coordinates for protein motions were extracted using Covariance matrices. These matrices were constructed using the backbone atoms of the protein (N, Cα, C) and were used to calculate the eigenvalues of maximum magnitude using GROMACS [78]. PyMOL was used to generate all the graphical representations [79].

## SUPPLEMENTARY MATERIALS

The supplementary materials can be found online with this article at https://doi.org/10.15302/J‐QB‐022‐0296.

## COMPLIANCE WITH ETHICS GUIDELINES

The authors Rashmi Panigrahi and Senthilkumar Kailasam declare that they have no conflict of interests.

All procedures performed in studies were in accordance with the ethical standards of the institution or practice at which the studies were conducted.

## Supporting information

Supplementary Information

Supplementary Information

Supplementary Information

Supplementary Information

Supplementary Information

Supplementary Information
