# Supplementary figures and images for: Mapping allosteric pathway in NIa‐Pro using computational approach
Source: Quant Biol. 2023 Mar 1;11(1):82–93. doi: 10.15302/J-QB-022-0296 (PMC12807009; doi:10.15302/J-QB-022-0296)

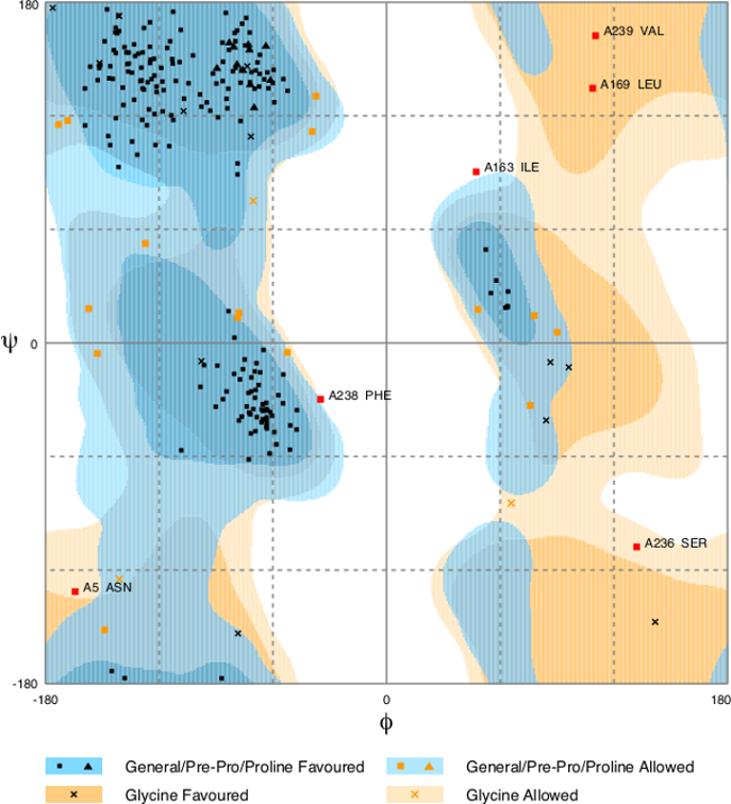

Supplement: Supplementary file 1 — Supplementary Information [file QUB2-11-82-s006.tiff]

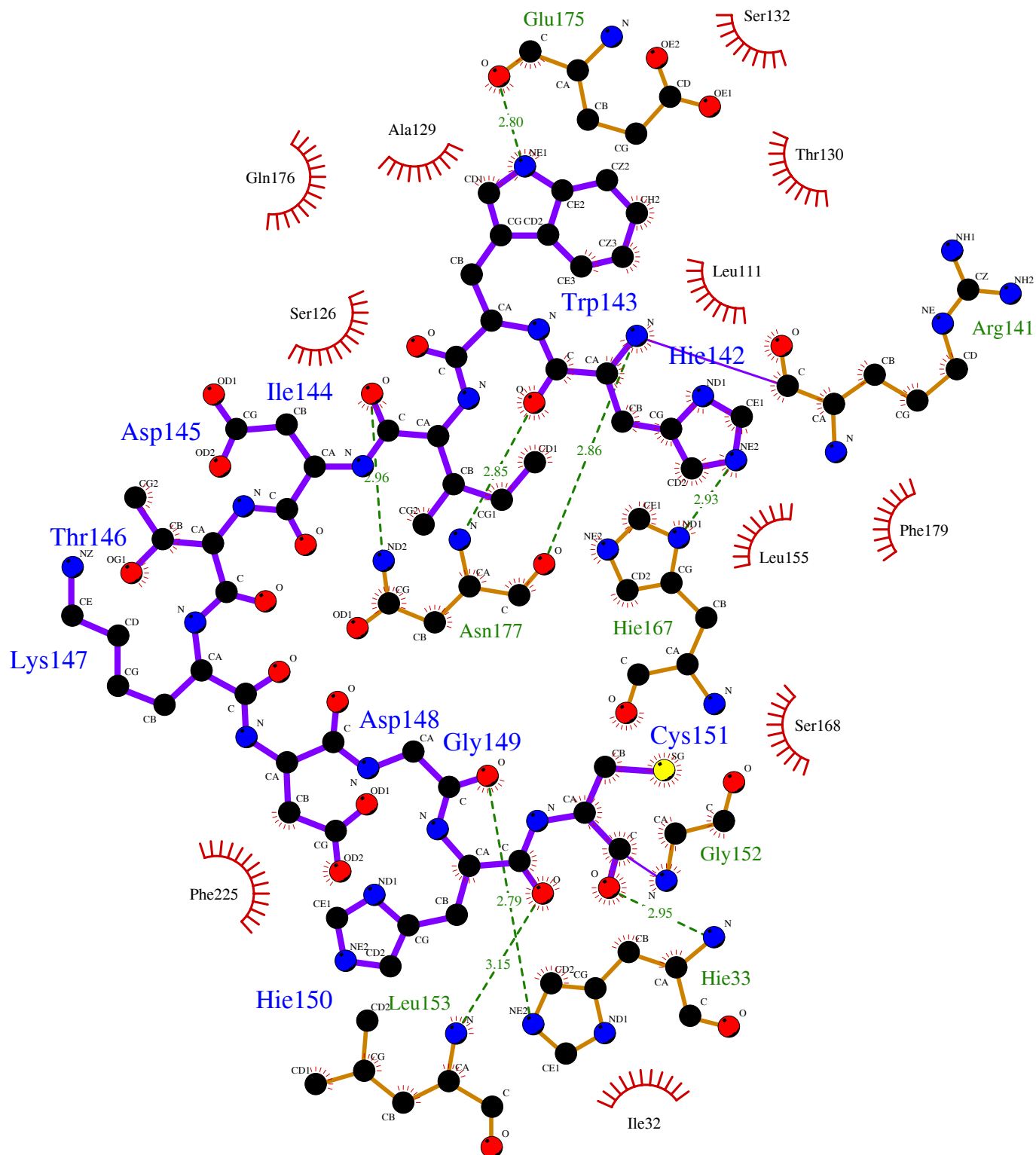

NIA\_S129\_average\_prot\_rst\_nowat

Supplement: Supplementary file 3 — Supplementary Information [file QUB2-11-82-s003.pdf]

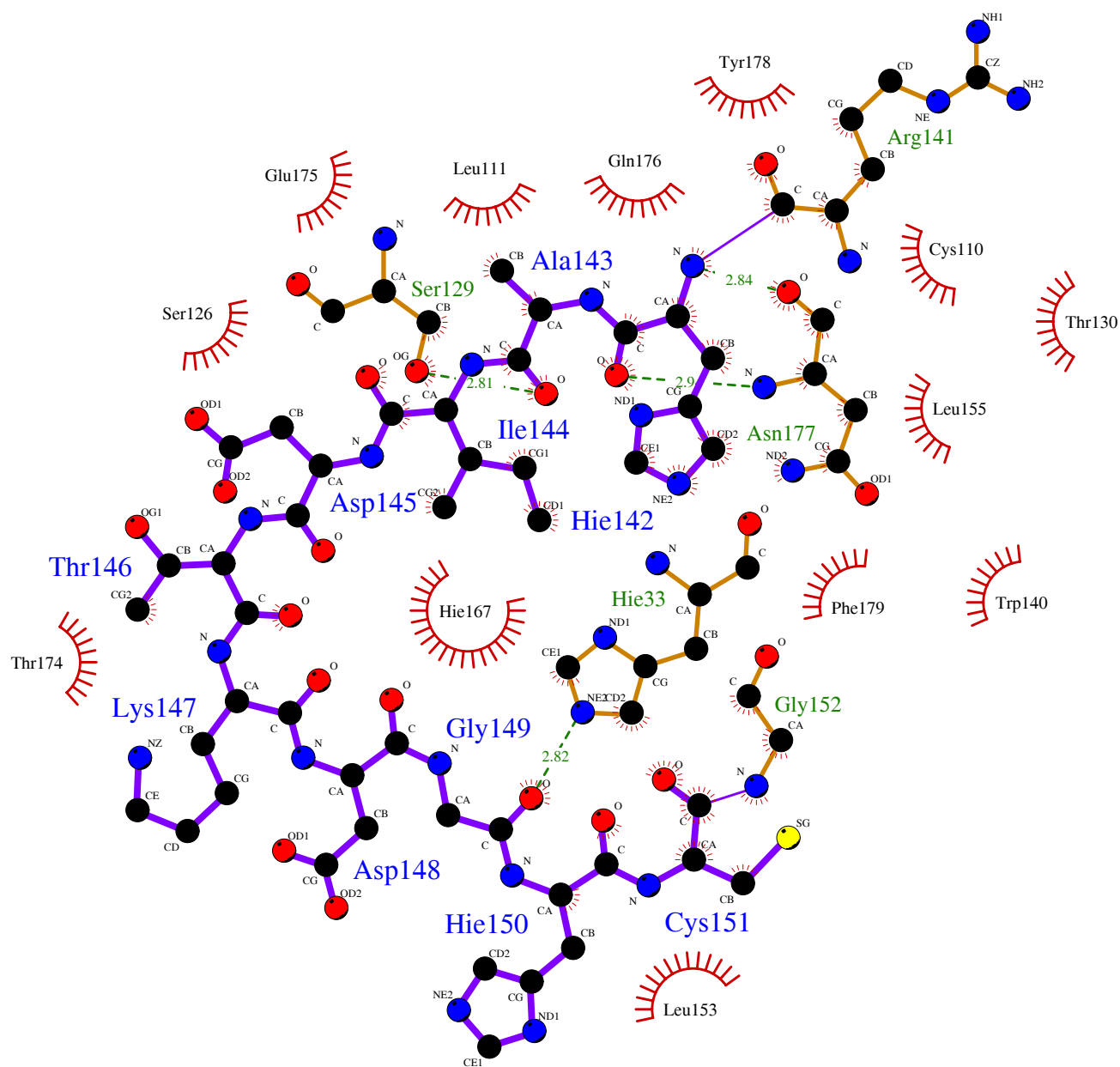

NIA\_W143\_average\_prot\_rst\_nowat

Supplement: Supplementary file 4 — Supplementary Information [file QUB2-11-82-s002.pdf]
