# Supplementary material for: Mapping allosteric pathway in NIa‐Pro using computational approach
Source: Quant Biol. 2023 Mar 1;11(1):82–93. doi: 10.15302/J-QB-022-0296 (PMC12807009; doi:10.15302/J-QB-022-0296)
Supplement: Supplementary file 6 — Supplementary Information [file QUB2-11-82-s005.pdf]

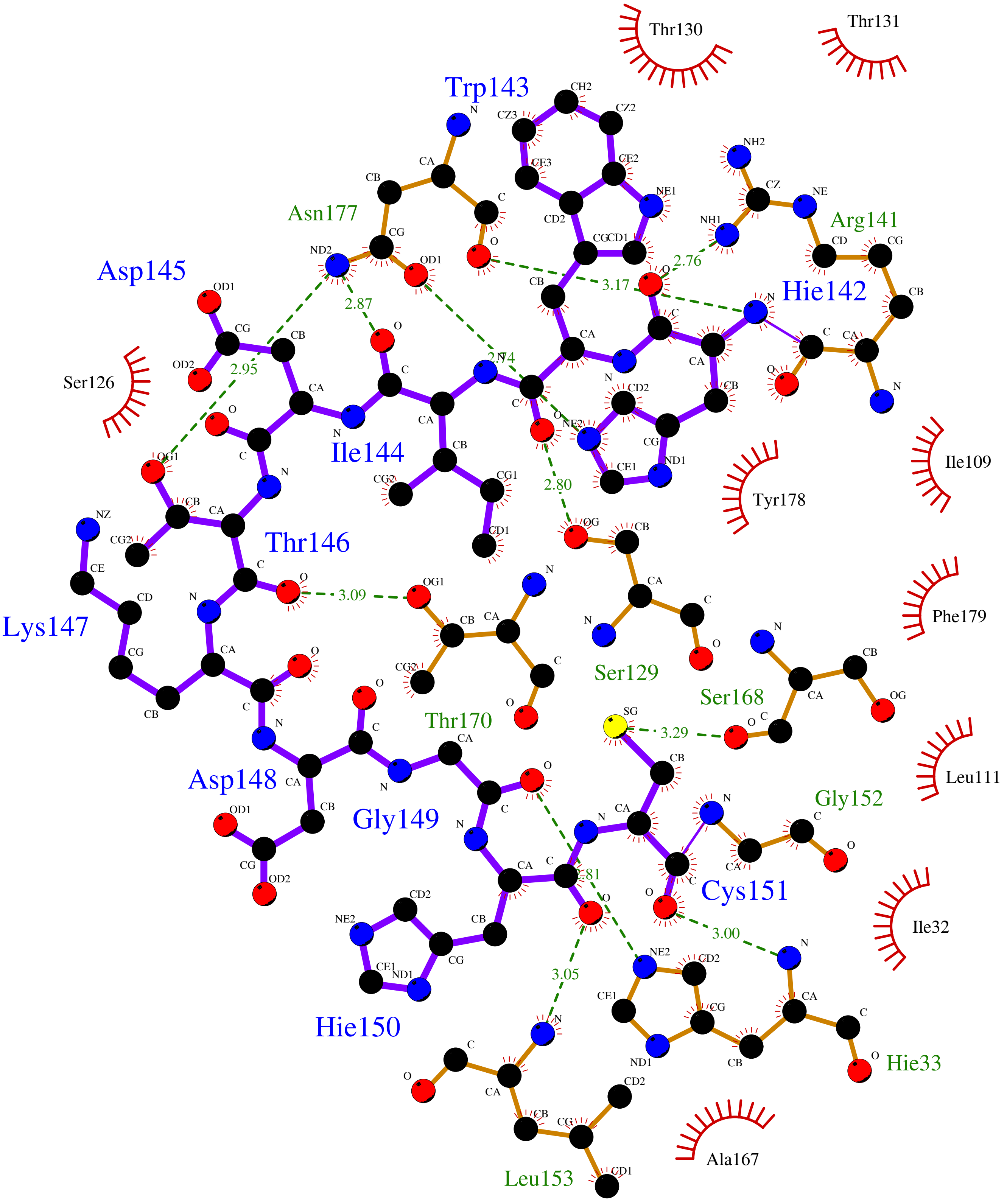

NIA_H167_average_prot_rst_nowat
